# Preoperative drug dispensing as predictor of surgical site infection.

**DOI:** 10.3201/eid0701.010110

**Published:** 2001

**Authors:** K S Kaye, K Sands, J G Donahue, K A Chan, P Fishman, R Platt

**Affiliations:** Beth Israel Deaconess Medical Center and Harvard Medical School, Boston, Massachusetts, USA. Kaye0001@mc.duke.edu

## Abstract

The system used by the National Nosocomial Infection Surveillance (NNIS) program to measure risk of surgical site infection uses a score of 3 on the American Society of Anesthesiologists (ASA)-physical status scale as a measure of underlying illness. The chronic disease score measures health status as a function of age, sex, and 29 chronic diseases, inferred from dispensing of prescription drugs. We studied the relationship between the chronic disease score and surgical site infection and whether the score can supplement the NNIS risk index. In a retrospective comparison of 191 patients with surgical site infection and 378 uninfected controls, the chronic disease score and ASA score were highly correlated. The chronic disease score improved prediction of infection by the NNIS risk index and augmented the ASA score for risk adjustment.


### Preoperative Drug Dispensing as Predictor of Surgical Site Infection

**Keith S. Kaye,* Kenneth Sands,* James G. Donahue,† K. Arnold Chan,† Paul Fishman,‡ and Richard Platt†**
*Beth Israel Deaconess Medical Center and Harvard Medical School, Boston, Massachusetts, USA; †Brigham and Women's Hospital and Harvard Medical School, Boston, Massachusetts, USA; ‡Group Health Cooperative of Puget Sound, Seattle, Washington, USA; and *†the Eastern Massachusetts CDC Prevention Epicenter, Boston, Massachusetts, USA

The system used by the National Nosocomial Infection Surveillance (NNIS) program to measure risk of surgical site infection uses a score of  3 on the American Society of Anesthesiologists (ASA)-physical status scale as a measure of underlying illness. The chronic disease score measures health status as a function of age, sex, and 29 chronic diseases, inferred from dispensing of prescription drugs. We studied the relationship between the chronic disease score and surgical site infection and whether the score can supplement the NNIS risk index. In a retrospective comparison of 191 patients with surgical site infection and 378 uninfected controls, the chronic disease score and ASA score were highly correlated. The chronic disease score improved prediction of infection by the NNIS risk index and augmented the ASA score for risk adjustment.

Approximately 325,000 surgical site infections occur each year in the United States, generating additional hospital costs in excess of $1 billion (1,2). Surgical site infection is also a major cause of increased hospital stay and death (3-6). Surgical site infection rates are an established measure of quality of clinical care (2,7), and reliable surveillance data are the foundation of effective infection control programs. However, to interpret surgical site infection surveillance rates, an effective risk adjustment system is needed. The National Nosocomial Infections Surveillance (NNIS) program uses a risk adjustment system for surgical site infection that includes three equally weighted variables: wound class, procedure duration, and the American Society of Anesthesiologists (ASA) score (8). The ASA score, a preoperative rating assigned to each patient, is a measure of the patient's general health status and coexisting conditions (9). Scores range from 1, representing a healthy person, to 5, representing a patient not expected to survive longer than 24 hours. The NNIS risk index assigns one point to patients with an ASA score ≥3. The ASA score is the only marker of coexisting conditions in the NNIS risk index.

Although the ASA score predicts surgical site infection, length of hospital stay, and risk for death (9-11), it is limited as a risk adjustment measure because of its subjectivity and poor inter-rater reliability (12-14). In a study in which 304 anesthesiologists assigned ASA scores to 10 hypothetical patients, the mean number of patients rated identically by the expert panel was 5.9 (13). The range of ASA scores is limited to five potential values; furthermore, there is often limited variation among patients undergoing similar procedures. Finally, the ASA score is not always available or easily accessible. It is typically not assigned for outpatient surgical procedures or procedures not attended by an anesthesiologist. An alternative rating system for coexisting diseases that does not have these limitations is needed.

A supplemental or alternative measure of a patient's underlying risk for surgical site infection is the chronic disease score, a measure

Address for correspondence: Keith S. Kaye, Duke University Medical Center, Box 3152, Durham, NC 27710; Fax: 919-684-3137; e-mail: Kaye0001@mc.duke.edu.

that predicts death, hospitalization, and use of health resources (15-17). For an individual patient, the chronic disease score is derived from the patient's age, sex, and presence or absence of each of 29 chronic diseases, which are inferred from ambulatory pharmacy dispensing records for the preceding 6 or 12 months.

We studied the relationships between surgical site infection and the chronic disease score and ASA score and evaluated whether the chronic disease score might improve or augment the NNIS risk index for surgical site infection.

### Methods

### Study Design

This nested case-control study involved cases of surgical site infection confirmed within 30 days after surgery, as well as individually matched controls. The study population was drawn from two patient cohorts (18,19) for which postoperative infection status and risk factors had been rigorously established. Data sources and methods of identifying surgical site infections have been described (18,19). We identified all adult members of the staff model component of Harvard Pilgrim Health Care (a mixed-model health maintenance organization) who underwent nonobstetric surgeries at Brigham and Women's Hospital from February 14, 1992, through March 6, 1993, and from May 19, 1997, through October 29, 1998. More than one surgery could be included for the same patient. Surgeries were excluded from analysis if they were performed on the same anatomic site within 3 months of prior surgery, on different sites but <1 month apart, or with infection as the indication for surgery. Patients were continuous members of the health-care plan for the 6 months before surgery and had prescription drug benefits. The NNIS classification system was used to categorize procedures on the basis of the International Classification of Diseases, 9th Edition (ICD-9) code (20). Surgical site infections occurring in the hospital or after discharge were identified by screening automated claims and electronic medical record data (18,19) and were confirmed by chart review according to NNIS criteria.

Controls were selected from patients who did not have a surgical site infection. Two controls were matched to each case from procedures performed within the following 6 weeks. In selecting controls, the greatest weight was given to matching procedure type (ICD-9 code, NNIS procedure, or NNIS procedure group), then to sex, age, and duration of procedure. The chronic disease score was calculated on the basis of prescriptions dispensed for the 6 months before surgery, available from an automated pharmacy record that captures essentially all pharmacy dispensing for health-plan members (17). Chronic disease scores have been reported on the basis of 6 or 12 months of pharmacy dispensing data. For this study, we used data from the 6 months before each patient's surgery, to minimize the number of exclusions caused by incomplete data. We used weights for disease classes derived for 12 months of data, because these provided a more current assessment of the importance of disease classes and because our emphasis was on the relative ranking of infected and uninfected patients, rather than on absolute risk prediction. We also computed the admission chronic disease score (a variant of the chronic disease score based solely on hospital pharmacy dispensing activity on the day of admission) by using an automated hospital database that captures all medications dispensed to hospitalized patients.

The ASA score, type of anesthesia administered, and emergency nature of the operative procedure were obtained by chart review. A procedure was considered an emergency if it was coded as such by the surgeon on the postoperative surgical summary sheets. Age, sex, procedure type, procedure duration, and wound class were obtained from automated databases.

### Data Analysis

The chronic disease score was studied as a continuous, ordinal, and dichotomous predictor. As an ordinal predictor, the score was divided into quartiles. All quartiles were entered into a conditional logistic regression model. The effects were plotted to determine if they had increased in a linear fashion.

Choosing a breakpoint to create a dichotomous chronic disease score variable was problematic because of the overfitting that would be introduced if the breakpoint were based on the result of a single conditional logistic regression model. Therefore, we generated 500 bootstrap samples, each the same size as the entire dataset, and selected by resampling from our entire dataset. Then, using conditional logistic regression to select among 50 candidate breakpoints after the data were controlled for age, sex, duration of surgery, and emergency surgery, we arrived at the best chronic disease score for each of the samples. A forward selection process was used to build the models; this process selected the chronic disease score, by producing 500 breakpoint values, of which the median value was chosen (21,22). In addition, the distribution of the 500 values was used to assess the stability and robustness of the final breakpoint. To determine a breakpoint for the admission chronic disease score, we followed the same procedure, except logistic regression was used rather than conditional logistic regression.

The ASA score was evaluated as both a 5-level ordinal variable, with values of 1,2,3,4, and 5, and as a dichotomous variable (ASA $\geq$3 and ASA <3, corresponding to the NNIS scoring system). The unadjusted relationship between chronic disease score and surgical site infection was analyzed by paired t test. All other analyses of the relationships between the chronic disease score and surgical site infection and between ASA and surgical site infection were performed by using conditional logistic regression. The unadjusted relationships between the admission chronic disease score and surgical site infection were analyzed with the Wilcoxon rank sum test and Fisher's exact test, because the cases of infection and their controls were no longer paired. Missing ASA scores (they were typically not available for ambulatory surgery procedures) were coded as dummy variables. Univariate relationships between other dichotomous variables and surgical site infection were analyzed with Cochran-Mantel-Haenszel summary statistics (for matched data) or Fisher's exact test (for unmatched data).

Relationships between continuous and ordinal variables were analyzed by conditional logistic regression for matched data and by Student's *t* test, Wilcoxon rank sum test, or logistic regression for unmatched data. The linearity assumption for continuous and ordinal predictors was examined in the same way as for the continuous chronic disease score. The relationships between the chronic disease score, the admission chronic disease score, and the ASA score were analyzed by Spearman correlation. For matched data, multivariate analyses assessing surgical site infection as the outcome were performed by conditional logistic regression. The following variables were included in the matched multivariate models: the chronic disease score, ASA score, type of anesthesia, emergency procedure vs. regular procedure, procedure duration, and wound class. The ability of these models to discriminate between infected and uninfected patients was compared by chi-square test to analyze differences in -2 log likelihood values.

Interaction terms between the chronic disease score and all other variables in the final model were evaluated and retained if they were statistically significant (p<0.05). For the unmatched analysis involving the admission chronic disease score, logistic regression was used. The following variables were included in the multivariate models: the admission chronic disease score, ASA score, sex, age, type of anesthesia, emergency procedure vs. regular procedure, procedure duration, and wound class. These regression models were analyzed for overfitting by the bootstrap method (1,000 bootstrap samples chosen as described).

An NNIS risk index score was calculated for each patient by assigning one point each for a contaminated wound, an ASA score $\geq$3, and surgical procedures lasting longer than the NNIS-derived 75th percentile for the duration of the procedure (23). Patients without an assigned ASA score were assumed to have a score <3, because more than two thirds of these surgeries were performed as outpatient procedures.

Two variant scores were also computed; in one of these, a chronic disease score $\geq$5,000 was substituted for ASA score $\geq$3, and in the other, a point was added to the NNIS score for a chronic disease score $\geq$5,000. The ability of these three scores to discriminate between infected and uninfected patients was compared by using unconditional logistic regression and the chi-square test to analyze differences in -2 log likelihood values. Unconditional regression was used to reduce the impact of our original selection process for uninfected controls. That process was influenced by procedure type and duration, both of which are part of the NNIS risk score. Analyses were performed with SAS software (SAS Institute, Cary, NC), system for Windows, v6.12).

### Results

The source population for cases and controls was 9,037 patients who underwent 10,457 operative procedures. One hundred ninety-six confirmed surgical site infections were identified (infection rate 2.1%), and 392 matched controls were selected. Drug dispensing data for the full 6 months before surgery were not available for 15 patients (5 cases and 10 controls), who were therefore excluded. If a case was excluded, its matched controls were also excluded (an additional 10 patients were excluded by this criterion).

The final study group included 191 cases and 372 controls (Table 1). The groups were comparable with regard to age (mean 51 years) and sex. General anesthesia (as opposed to other types of anesthesia) was associated with surgical site infection (Table 1). Although procedure duration was included in the multivariate matching process, longer duration was associated with surgical site infection. Procedures classified as emergency had borderline association. Other recognized risk factors, such as procedure type and NNIS wound class, were not significantly associated with infection because of the matching procedure that was used to select uninfected controls. ASA score was associated with infection, but chronic disease score was marginally associated.

The relationships were calculated between surgical site infection rates and ASA scores for the 80% of patients for whom scores had been assigned and between surgical site infection rates and the chronic disease scores (Figure 1). Patients with missing ASA scores were included in the chronic disease score groupings. The risk for surgical site infection increased with ASA group scores. In this group of patients (selected so that one third had a surgical site infection) 27.8% of those with an ASA score of 1, representing 25% of the study population, had surgical site infections, while 61.5% of those with scores of 4, representing 3% of the study population, had infections. No patients had scores of 5.

For the chronic disease score groups, the proportion with surgical site infections also

Table 1. Characteristics of the study population

| Variable | Cases (n=191) | Controls (n=372) | p value | Odds ratio (95% CI) |
|---|---|---|---|---|
| Mean age (yrs) | 51.2 | 51.5 | NA | NA |
| Male sex (%) | 91 (47.6) | 182 (48.9) | NA | NA |
| NNIS wound class, contaminated or infected (%) | 8 (4.2) | 13 (3.5) | NA | NA |
| Surgical speciality (%) | | | NA | NA |
| General | 54 (28.3) | 107 (28.8) | | |
| Cardiac | 41 (21.5) | 78 (21.0) | | |
| Orthopedic | 31 (16.2) | 63 (16.9) | | |
| Plastic | 17 (8.9) | 30 (8.1) | | |
| Urologic | 14 (7.3) | 27 (7.3) | | |
| Vascular | 10 (5.2) | 19 (5.1) | | |
| Gynecologic | 5 (2.6) | 10 (2.7) | | |
| Neurosurgical | 4 (2.1) | 8 (2.2) | | |
| Other | 15 (7.9) | 30 (7.8) | | |
| General anesthesia (%) | 132 (69.1) | 222 (59.7) | 0.004 | 2.19 (1.29,3.72) |
| Duration of surgery, minutes (IQR) | 105 (55-211) | 83 (40-154) | <0.001[a] | 1.75 (1.34,2,29)[a] |
| Emergent procedure (%) | 11 (5.8) | 10 (2.7) | 0.07 | 2.21 (0.94,5.21) |
| ASA score (%) | | | 0.002[b] | 2.0 (1.4, 2.9)[b] |
| Missing | 30 (15.7) | 80 (21.5) | | |
| 1 | 32 (16.8) | 83 (22.3) | | |
| 2 | 62 (32.5) | 130 (34.9) | | |
| 3 | 59 (30.9) | 74 (19.9) | | |
| 4 | 8 (4.2) | 5 (1.4) | | |
| 5 | 0 | 0 | | |
| Chronic disease score | | | | |
| Median (IQR) | 2,219 (1,101-5,673) | 1,641 (809-3,588) | 0.09 | NA |

[a]Values are for duration as a 6-level ordinal variable. Risk is per-unit increase in duration category.
[b]ASA as a four-level ordinal variable, excluding missing values. Risk is per-unit increase in ASA category, missing values excluded.
OR = odds ratio.
ASA = American Society of Anesthesiologists physical status scale; IQR = Interquartile range.

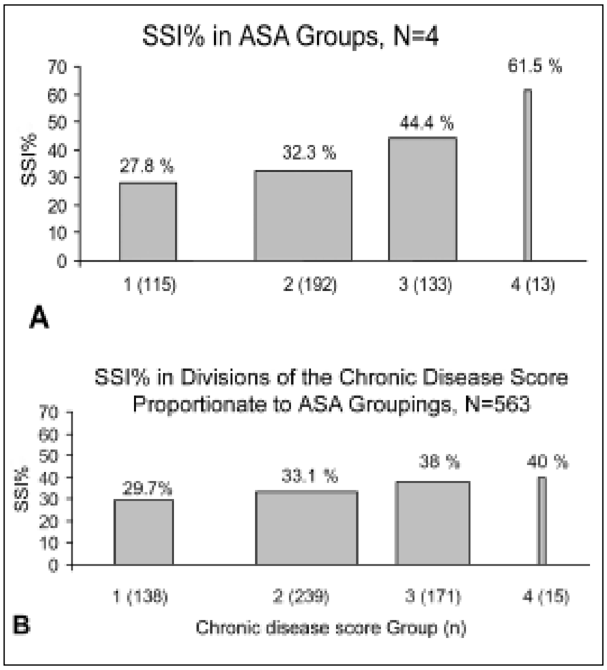

Figure 1 (A and B). Risk for surgical site infection in groups proportionate to ASA groupings for both the American Society of Anesthesiologists (ASA)-physical status score (A) and chronic disease score (B). The width of each bar is proportional to the sample size in that particular group. The percentage above each bar represents the proportion of persons in the group with infection.

increased with the chronic disease group scores. The group with the lowest 25% of the chronic disease scores had a surgical site infection proportion of 29.7%, while those with the highest 3% of scores had a surgical site infection proportion of 40%. The ASA score and the chronic disease score were strongly correlated (r=0.58, p<0.001). One major breakpoint in surgical site infection rates occurred between the lower 80% the upper 20% of the chronic disease score values, as determined by conditional logistic regression. Therefore, the chronic disease score was analyzed as a dichotomous variable, using a breakpoint of chronic disease scores ≥5,000 and <5000.

Relationships were calculated between surgical site infection and chronic disease scores >5,000 and between surgical site infection and ASA scores (Table 2a). In the unadjusted analysis (but with data controlled for the original matching variables of age, sex, and procedure type), both the chronic disease score ≥5,000 and ASA ≥3 were strong predictors of surgical site infection (for chronic disease score, odds ratio [OR] 2.6, 95% confidence interval [CI] 1.6-4.2, p<0.001; for ASA, OR 3.1, 95% CI 1.7-5.5, p<0.001). When added to a multivariate model

Table 2.

a. Relationships between chronic disease score, ASA score, and surgical site infection

| | Cases | Controls | Unadjusted[a] | | Adjusted[b] | |
|---|---|---|---|---|---|---|
| Variable | (n-191) | (n=372) | p value | OR (95% CI) | p value | OR (95% CI) |
| Chronic disease score ≥5,000 (%) | 55 (28.8) | 60 (16.1) | <0.001 | 2.6 (1.6-4.2) | 0.001 | 2.6 (1.5-4.7) |
| ASA ≥3 (%) | 67(35.1)[c] | 79 (21.2)[d] | <0.001 | 3.1 (1.7-5.5) | 0.03 | 2.0 (1.1-3.7) |

[a]Unadjusted values are controlled for age, sex, and procedure duration because of the matching process used to select uninfected subjects.
[b]Adjusted for chronic disease score ≥5,000, ASA ≥3, type of anesthesia, emergent nature of the procedure, procedure duration (ordinal), and wound class.
[c]Thirty (15.7%) cases had missing ASA scores.
[d]Eighty-two (21.7%) controls had missing ASA scores.
ASA = American Society of Anesthesiologists; OR = odds ratio; CI = confidence intervals b. Relationships between admission chronic disease score, chronic disease score, ASA score and surgical site infection

| | Cases | Controls | Unadjusted | | Adjusted[a] | |
|---|---|---|---|---|---|---|
| Variable | (n=51) | (n=67) | p value | OR (95% CI) | p value | OR (95% CI) |
| Admission chronic disease score ≥4,500 (%) | 18 (35) | 5 (7) | <0.001 | 6.8 (2.3-19.9) | 0.003 | 6.2 (1.9-20.4) |
| Chronic disease score ≥5,000 (%) | 22 (43) | 17 (25) | 0.05 | 2.2 (1.0-4.9) | 0.32 | 1.6 (0.6-3.9) |
| ASA ≥3 (%) | 39 (83)[b] | 36 (58)[c] | 0.007 | 3.5 (1.4-8.8) | 0.03 | 3.4 (1.1-10.2) |

[a]Adjusted for sex, age, type of anesthesia, emergency nature of the procedure, procedure duration (ordinal) and wound class.
[b]Four cases had missing ASA scores.
[c]Five controls had missing ASA scores.

that already included the ASA score and other factors associated with surgical site infection, the chronic disease score improved the explanatory value of the model ($p<0.001$). In this model, both ASA and the chronic disease score remained significant predictors of surgical site infection (chronic disease score $\geq$5000, OR 2.6, 95% CI 1.5-4.7, p=0.001; ASA $\geq$3, OR 2.0, 95% CI 1.1-3.7, p=0.03).

A chronic disease score based solely on admission medications (admission chronic disease score) was also studied (Table 2b). From 191 total cases of surgical site infection and 372 uninfected controls, cases with admission and surgery on the same day were excluded, leaving 51 cases and 67 controls. The median admission chronic disease score for cases was 2,218 (interquartile range [IQR] 1,285-4,818) and for controls was 1,285 (IQR 1,209-2,729) (p=0.008, Wilcoxon rank sum test). The admission chronic disease score was correlated with the 6-month chronic disease score ($p<0.001$, r=0.45) and with the ASA score (p=0.02, r=0.22). The admission chronic disease score was analyzed as a dichotomous variable, using a breakpoint of chronic disease score $\geq$4,500 and chronic disease score <4,500. Admission chronic disease score $\geq$4,500, chronic disease score $\geq$5,000 and ASA $\geq$3 were all associated with surgical site infection; the association was strongest for the admission chronic disease score (for admission chronic disease score, OR 6.8, $p<0.001$; for the 6-month chronic disease score, OR 2.2, p=0.05; for ASA, OR 3.5, p=0.007).

After the data were controlled for anesthesia type, emergency nature of surgery, sex, age, procedure duration, and wound class by logistic regression, the model that included the admission chronic disease score had better predictive value for surgical site infection than the model containing the chronic disease score based on 6 months of preoperative medications ($p<0.01$) and the model that included the ASA score ($p<0.03$). In these multivariate models, the admission chronic disease score was a stronger predictor of surgical site infection than the chronic disease score based on 6 months of preoperative medications and the ASA score (for the admission chronic disease score, OR 6.2, p=0.003; for the 6-month chronic disease score, OR 1.6, p=0.32; for ASA score, OR 3.4, p=0.03); the ASA score did not improve the model already containing the admission chronic disease score (p=0.1).

In this population, the NNIS risk index was correlated with infection status (Figure 2A). As the risk index score increased, the proportion of infected patients increased (27% for a risk score of 0, 100% for a risk score of 3). Both the risk index that substituted the chronic disease score for the ASA score (Figure 2B) and the risk index that added the chronic disease score to the conventional NNIS score (Figure 2C) increased progressively in the proportion infected in each risk class. After the data were controlled for type

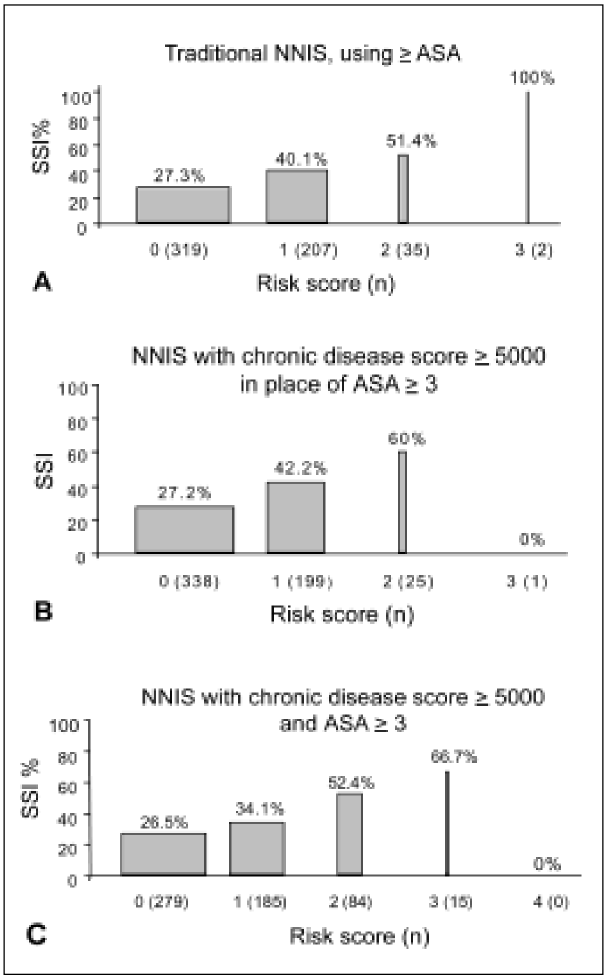

Figure 2 (A, B, and C). Risk of surgical site infection in different risk index categories. The width of each bar is proportional to the sample size in that particular group; A) shows the traditional National Nosocomial Infection Surveillance (NNIS) risk index categories; B) shows a modified NNIS risk index with chronic disease score $\geq$5,000 substituted for ASA $\geq$3; C) shows a modified NNIS risk index, incorporating both chronic disease score $\geq$5,000 and the traditional NNIS risk index categories. In each group, the percentage of patients with infections is shown.

of anesthesia used and emergency nature of the surgery, the risk index that included a chronic disease score ≥5,000 in place of ASA ≥3 (Figure 2B) was a stronger predictor of surgical site infection than the traditional NNIS score (Figure 2A) ($p<0.05$). The risk index that included both a chronic disease score ≥5,000 and an ASA ≥3 (Figure 2C) was a better predictor of surgical site infection than both the traditional risk score ($p<0.001$) and the score that substituted the chronic disease score for an ASA ≥3 ($p<0.01$).

### Conclusions

The chronic disease score, like the ASA score when other known risk factors for infection were taken into consideration, was a strong predictor of postoperative surgical site infection. The strength of the association between the ASA score and the chronic disease score was impressive since the two scores are derived by completely different methods. The chronic disease score performed comparably with the ASA score as a component of the NNIS risk index, and an NNIS-like index based on both the chronic disease and ASA scores performed better than the conventional NNIS index. In our limited sample of patients who were admitted to the hospital before the day of surgery, a chronic disease score based solely on medications dispensed on the day of admission was a stronger predictor of surgical site infection than either the ASA or 6-month chronic disease scores. Thus, either the 6-month or admission chronic disease scores might be considered as an alternative to the ASA score and may provide better risk stratification.

Compared with the ASA score, the chronic disease score has several potential advantages as a risk-adjuster for surgical site infection. It is objective and, since it can be calculated from the administrative databases of health insurance organizations, sometimes more readily available than the ASA score. Pharmacy databases are often the most complete and accurate ones maintained by health plans, and the information in these systems is usually easy to interpret. In addition, pharmacy data can often be obtained from third parties, such as pharmacy benefit managers, or directly from the pharmacies where prescriptions were filled.

Since the chronic disease score can be derived from electronic pharmacy data, it lends itself to use as a risk-adjuster in automated systems for monitoring surgical site infection, particularly in patients discharged from the hospital who do not return to the hospital for care. Such systems would be applicable not only for the surveillance and risk adjustment of surgical site infection rates for individual hospitals, but also for larger populations such as members of a health maintenance organization or other health insurance group. The chronic disease score based solely on admission medications might be attractive for use in hospital-based surveillance systems if the results in this study are reproducible in larger patient populations.

This study had some limitations. Since age is a component of the chronic disease score and cases were matched to controls by age, the observed association between the chronic disease score and surgical site infection may be underestimated. In addition, because components of the NNIS risk index were used to match uninfected controls to cases, our results may underestimate the associations between surgical site infection and the traditional NNIS risk index, the risk index using the chronic disease score in place of an ASA score, and the index incorporating both the chronic disease and ASA scores. Additionally, all the procedures were performed at a single hospital, increasing the likelihood that ASA scores were assigned more consistently than in the population at large. Such an effect would have overestimated the utility of the ASA score. Restriction to a single institution may also affect the generalizability of these results. Although we do not have a ready explanation for ways in which the chronic disease score could have a special relationship to infection in this population, our findings should be tested in other settings. Our relatively limited sample size precludes investigation of whether the chronic disease score performed differently for different types of procedures or specific groups of patients. Validation of our model in a larger population with a more diverse set of procedure types would be worthwhile. Finally, the chronic disease score can only be assessed readily for patients whose pharmacy dispensing data are available. This information exists for most patients through pharmacy benefit systems, but few hospitals have ready access to this information.

Aspects of the association between chronic disease score and surgical site infection that warrant additional exploration include assessing the value of the chronic disease score, either alone or in addition to the ASA score for specific procedure types, and developing new weights for the chronic disease categories to improve predictive value. Evaluation of a chronic disease score based on admission medications using a larger sample size than the one used in this study and including patients from multiple institutions would be of interest.

In summary, the chronic disease score provides a useful risk-adjuster for surgical site infection. It is objective and can often be obtained from automated information available from standard health insurance claims data. It may be available when the ASA score is not, so it could be used either in place of the ASA score or in addition to it. Further investigation of the chronic disease score and its association with surgical site infection is warranted.

### Acknowledgments

We thank E. John Orav for his expert statistical guidance.

This study was presented in part at the Society for Healthcare Epidemiology of America annual meeting, San Francisco, California, April 1999, and at the 4th Decennial International Conference on Nosocomial and Health Care-Associated Infections, Atlanta, Georgia, March 2000.

This work was supported by the Harvard Pilgrim Health Care Foundation and Cooperative Agreement UR8/ CCU115079 from the Centers for Disease Control and Prevention.

Dr. Kaye is the associate hospital epidemologist and an infectious disease attending physician at Duke University Medical Center. His professional interests include hospital epidemiology and efficient animicrobial drug utilization. His academic interests focus on the study of outcomes and risk factors associated with multidrug-resistant bacterial pathogens and surgical site infections.

### References

1. Mayhall CG. Surgical infections, including burns. In: Wenzel RP, editor. Prevention and control of nosocomial infections. 2nd ed. Baltimore: Williams & Wilkins; 1993:p. 614-64.
2. Wenzel RP. Nosocomial infections, diagnosis-related groups, and study on the efficacy of nosocomial infection control: economic implications for hospitals under the prospective payment system. Am J Med 1985;78:3-7.
3. Cruse PJ, Foord R. The epidemiology of wound infection: a 10-year prospective study of 62,939 wounds. Surg Clin North Am 1980;60:27-40.
4. Cruse P. Wound infection surveillance. Rev Infect Dis 1981;3:734-7.
5. Boyce JM, Potter-Bynoe G, Dziobek L. Hospital reimbursement patterns among patients with surgical wound infections following open heart surgery. Infect Control Hosp Epidemiol 1990;11:89-93.
6. Poulsen KB, Bremmelgaard A, Sorensen AI, Raahave D, Petersen JV. Estimated costs of postoperative wound infections: a case-control study of marginal hospital and social security costs. Epidemiol Infect 1994;113:283-95.
7. Haley RW, Culver DH, White JW, Morgan WM, Emori TG, Munn VP, et al. The efficacy of infection surveillance and control programs in preventing nosocomial infections in US hospitals. Am J Epidemiol 1985;121:182-205.
8. Centers for Disease Control and Prevention. Draft guidelines for the prevention of surgical site infection, 1998. Federal Registister 1998;63:33168-92.
9. Cullen DJ, Apolone G, Greenfield S, Guadagnoli E, Cleary P. ASA Physical Status and age predict morbidity after three surgical procedures [comments]. Ann Surg 1994;220:3-9.
10. Vacanti CJ, VanHouten RJ, Hill RC. A statistical analysis of the relationship of physical status to postoperative mortality in 68,388 cases. Anesth Analg 1970;49:564-6.
11. Garibaldi RA, Cushing D, Lerer T. Risk factors for postoperative infection. Am J Med 1991;91:158S-163S.
12. Haynes SR, Lawler PG. An assessment of the consistency of ASA physical status classification allocation [see comments]. Anaesthesia 1995;50:195-9.
13. Owens WD, Felts JA, Spitznagel EL Jr. ASA physical status classifications: a study of consistency of ratings. Anesthesiology 1978;49:239-43.
14. Salemi C, Anderson D, Flores D. American Society of Anesthesiology scoring discrepancies affecting the National Nosocomial Infection Surveillance System: surgical-site-infection risk index rates. Infect Control Hosp Epidemiol 1997;18:246-7.
15. Von Korff M, Wagner EH, Saunders K. A chronic disease score from automated pharmacy data. J Clin Epidemiol 1992;45:197-203.
16. Fishman P, Goodman M, Hornbrook M, Meenan R, Bachman D, O'Keefe-Rosetti M. Risk adjustment using automated using pharmacy data: a global chronic disease score. 2nd International Health Economic Conference, June 8, 1999, Rotterdam, the Netherlands, 1999.
17. Clark DO, Von Korff M, Saunders K, Baluch WM, Simon GE. A chronic disease score with empirically derived weights. Med Care 1995;33:783-95.
18. Sands K, Vineyard G, Platt R. Surgical site infections occurring after hospital discharge. J Infect Dis 1996;173:963-70.

19. Sands K, Vineyard G, Livingston J, Christiansen C, Platt R. Efficient identification of postdischarge surgical site infections: use of automated pharmacy dispensing information, administrative data, and medical record information. J Infect Dis 1999;179:434-41.
20. Emori TG, Culver DH, Horan TC, Jarvis WR, White JW, Olson DR, et al. National nosocomial infections surveillance system (NNIS): description of surveillance methods. Am J Infect Control 1991;19:19-35.
21. Hosmer DW, Lemeshow S. Applied logistic regression. New York: John Wiley & Sons; 1989.
22. Efron B, Tibshirani RJ. An introduction to the bootstrap. New York: Chapman & Hall; 1993.
23. Culver DH, Horan TC, Gaynes RP, Martone WJ, Jarvis WR, Emori TG, et al. Surgical wound infection rates by wound class, operative procedure, and patient risk index. National Nosocomial Infections Surveillance System. Am J Med 1991;91:152S-7S.
